# *Drosophila Melanogaster* as a Model System for Studies of Islet Amyloid Polypeptide Aggregation

**DOI:** 10.1371/journal.pone.0020221

**Published:** 2011-06-14

**Authors:** Sebastian Wolfgang Schultz, K. Peter R. Nilsson, Gunilla Torstensdotter Westermark

**Affiliations:** 1 Department of Clinical and Experimental Medicine, Linköping University, Linköping, Sweden; 2 IFM-Department of Chemistry, Linköping University, Linköping, Sweden; 3 Department of Medical Cell Biology, Uppsala University, Uppsala, Sweden; Alexander Flemming Biomedical Sciences Research Center, Greece

## Abstract

**Background:**

Recent research supports that aggregation of islet amyloid polypeptide (IAPP) leads to cell death and this makes islet amyloid a plausible cause for the reduction of beta cell mass, demonstrated in patients with type 2 diabetes. IAPP is produced by the beta cells as a prohormone, and proIAPP is processed into IAPP by the prohormone convertases PC1/3 and PC2 in the secretory granules. Little is known about the pathogenesis for islet amyloid and which intracellular mechanisms are involved in amyloidogenesis and induction of cell death.

**Methodology/Principal Findings:**

We have established expression of human proIAPP (hproIAPP), human IAPP (hIAPP) and the non-amyloidogenic mouse IAPP (mIAPP) in *Drosophila melanogaster*, and compared survival of flies with the expression driven to different cell populations. Only flies expressing hproIAPP in neurons driven by the Gal4 driver elav^C155,Gal4^ showed a reduction in lifespan whereas neither expression of hIAPP or mIAPP influenced survival. Both hIAPP and hproIAPP expression caused formation of aggregates in CNS and fat body region, and these aggregates were both stained by the dyes Congo red and pFTAA, both known to detect amyloid. Also, the morphology of the highly organized protein granules that developed in the fat body of the head in hIAPP and hproIAPP expressing flies was characterized, and determined to consist of 15.8 nm thick pentagonal rod-like structures.

**Conclusions/Significance:**

These findings point to a potential for *Drosophila melanogaster* to serve as a model system for studies of hproIAPP and hIAPP expression with subsequent aggregation and developed pathology.

## Introduction

Today, there are 27 proteins that have been identified as main component of amyloid deposits in human [1]. Of these proteins, 14 form amyloid fibrils with a deposition restricted to a single tissue or organ, and these diseases are referred to as localized amyloidosis. There is no biochemical relationship between these amyloid proteins, but interestingly, five of those are polypeptide hormones, recognized to be stored in the secretory granules at high concentration, a known risk factor for aggregation. Some of the local forms of amyloid diseases are connected to severe maladies, such as type 2 diabetes where IAPP misfolds and deposits as amyloid in the islet of Langerhans [2], [3], and Alzheimer's disease where the Aβ protein is deposited in the CNS [4].

In recent years, the role for hIAPP in the development of type 2 diabetes has gained a lot of attention. In particular, the formation of cell toxic oligomers as a cause for the observed reduction of insulin producing cells, detected in these patients are believed to be important [5]. IAPP is produced by the beta cells [6] and co-secreted with insulin upon stimulation [7]. IAPP acts as a modulator for insulin release and reduces voltage-gated calcium channel activation and insulin secretion [8]. IAPP is synthesized as a 67 residues prohormone, and undergoes posttranslational processing to become biologically active. This is performed by the prohormone convertases PC1/3 and PC2 and takes place in the secretory granules [9]. Deficiency in processing of hproIAPP into hIAPP is associated with an increase in amyloidogeneity [10]. Expression of human preproIAPP in cell lines deficient of PC2 (GH3, American type culture collection, Manassas, VA) and/or PC1/3 (GH4C1, AtT-20, American type culture collection) increases the risk for amyloid formation [11] and cell death by apoptosis [12]. It was also shown that hproIAPP and hIAPP both spontaneously form amyloid like fibrils *in vitro* [13], [14]. Little is known about the mechanisms that cause a native protein to unfold and misfold into amyloid [15]. Islet amyloid is present to some degree in almost all individuals with type 2 diabetes [16], [17]. The amyloid load correlates to some degree with the severity of the diabetes condition in that patients that demand insulin for treatment of their disease have more amyloid deposited [18]. In Asian populations a single point mutation in the IAPP gene (IAPPS20G) [19] has been linked to an increased risk to develop type 2 diabetes [20]. Islet amyloid develops also in old cats [21] and in non-human primates [22], [23]. In baboons the amount of islet amyloid correlates with fasting plasma glucose levels [24]. Mouse and rat do not develop islet amyloid; this is a difference that depends on the amino acid sequence and especially three proline residues present in the region 20–29 of rodent IAPP are thought to be responsible for this [25]. Therefore, different transgenic strains that express human IAPP have been created. Studies performed with these animals support the importance of islet amyloid for development of diabetes [26], [27].

The present work aims to establish a new *in vivo* model for expression of IAPP or proIAPP that could be used for elucidation of intracellular events triggered by protein aggregation. We used *Drosophila melanogaster* as model system which is already established for several other amyloid related diseases, such as Alzheimer's disease [28], familial amyloidotic polyneuropathy (FAP) [29], [30], and the prion disease Gerstmann-Sträussler-Scheinker syndrome [31].

Herein, we describe that hproIAPP expression reduces longevity of *Drosophila* when expressed in the CNS, while longevity is not influenced by hIAPP or mIAPP expression. Furthermore, we were able to demonstrate that both hproIAPP and hIAPP are successfully secreted from the neurons, and assemble into aggregates with amyloid tinctorial characteristics. After secretion the peptides can be taken up by cells in the fat body of the head. In these cells the proteins form highly ordered protein aggregates with a rod-like structure.

## Results and Discussion

### Transgenic *Drosophila melanogaster*

In human, IAPP together with calcitonin [32], calcitonin gene related peptide (CGRP) [33], adrenomedullin [34], and intermedin/adrenomedullin 2 [35] are all members of the calcitonin gene family that are important for regulation of a diversity of physiological functions. The *Drosophila melanogaster* genome was searched for nucleotide or amino acid sequences similar to any of these five polypeptide hormones, but without any success.

**Figure 1:**
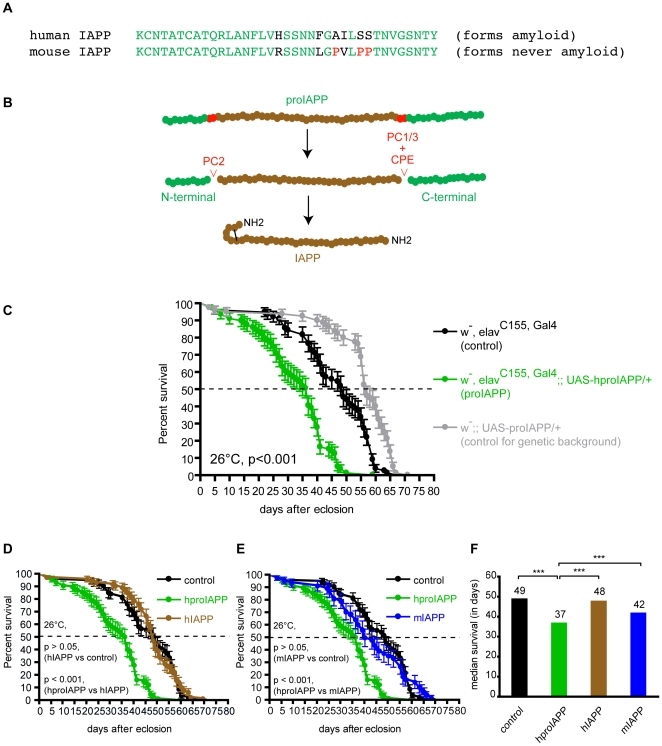
One letter code for human and mouse IAPP residues 1–37. Amino acid identity is shown in green and residue substitutions are shown in black, except for the three proline residues substitutions present in mouse IAPP that are shown in red (A). Proline residues are known beta-breakers and it is believed that these residues hinder IAPP-aggregation. ProIAPP processing. In man, proIAPP is synthesised as a 67 residue long polypeptide that undergoes posttranslational cleavage at dibasic residues (red) performed by the prohormone convertases PC2 and PC1/3. The two basic residues that remain at the C-terminus after PC1/3 processing are removed by carboxypeptidase E. Thereafter, the remaining C-terminal glycine residue is used for C-terminal amidation. A disulphide-bond is formed between residues 2 and 7 (B). Survival curve for flies expressing hproIAPP shows a reduction in life span. Expression of proIAPP was directed to neurons by the elav^C155,Gal4^ driver (C). This expression resulted in a significant reduction of lifespan (green), when compared to control flies (elav^C155,Gal4^, black) or flies containing *UAS-proIAPP* only (grey). These latter flies contain the inserted DNA, but do not express the corresponding protein due to absence of Gal4. (D) Comparison of the survival of hIAPP (brown) and elav ^C155,Gal4^, (black) expressing flies shows that hIAPP expression does not reduce the survival (p>0.05). The hproIAPP (green) expressing flies showed a significant shorter lifespan than hIAPP (brown, p<0.001). (E) Comparison of the survival of mIAPP (blue) with control flies elav^C155, Gal4^, (black) showed that mIAPP expression does not reduce the survival of the flies (p>0.05). Flies expressing mIAPP (blue) lived longer than hproIAPP expressing flies (green, p<0.001). (F) The median survival for control flies (black), hproIAPP (green), hIAPP (brown) and mIAPP (blue) are 49, 37, 48 and 42 days, respectively, and the survival of hproIAPP flies is significantly reduced when compared to flies from the control, hIAPP and mIAPP strains (p<0.001).The survival for hIAPP flies is not reduced when compared to mIAPP or control flies. The survival studies were performed in an incubator with 70% humidity, at 26°C.

A large number of transgenic strains expressing hproIAPP, hIAPP or mIAPP were successfully established, and the expected DNA sequences of the different transgenes were verified after sequencing. The amino acid sequence for hIAPP and mIAPP are presented in Figure 1 A, and the posttranslational processing sites for hproIAPP are presented in Figure 1 B. The survival of the three hproIAPP lines hproIAPP#14.2, hproIAPP#18.5 and hproIAPP#20.4 were compared to the survival of hemizygous Gal4 expressing control flies, and all three hproIAPP transgenic flies showed reduced survival when compared to the hemizygous Gal4 control flies. HproIAPP#14.2 and hproIAPP#18.5 had significantly reduced survival with p-values of <0.0001 and 0.0175, while flies from line 20.4 did not reach a significant reduction (p-value = 0.0577) (Figure S1). The relative amounts of hproIAPP mRNA was determined by QT-PCR. When the values were normalized against the mRNA levels for hproIAPP#14.2, the levels for hproIAPP#18.5 and hproIAPP#20.4 lines were determined to be 249% and 128%, respectively. A double transgenic hproIAPP expressing fly were established by crossing hproIAPP#20.4 and hproIAPP#14.2 flies. Survival analysis of this double hproIAPP fly line showed a reduced survival (p<0.0001) compared to hemizygous Gal4 expressing control flies. Survival analysis were also carried out on four hIAPP lines and four mIAPP lines, and neither of these lines revealed any reduction in survival when compared to hemizygous Gal4 expressing control flies. The mRNA expression levels for the hIAPP lines were normalized against hIAPP#6 and determined to be 42% in hIAPP#1, 89% in hIAPP#2 and 110% in hIAPP#5 flies. The mRNA expression levels for mIAPP were normalized against the mIAPP#9 and determined to be 435% in mIAPP#1, 110% in mIAPP#2 and 170% in mIAPP#3 flies.

Since the mRNA expression levels of hIAPP and mIAPP did not affect the survival the transgenic lines hIAPP#6, mIAPP#9 and hproIAPP#14.2 were selected for the further work. All lines had red eyes with the same intensity.

The expression levels for the hproIAPP#14.2, hIAPP#6 and mIAPP#9 transgenes were analyzed by QT-PCR, and levels for hIAPP and mIAPP were determined to be 72% and 62% of hproIAPP, respectively.

### Generation of transgene expressing flies and control flies

**Figure 2:**
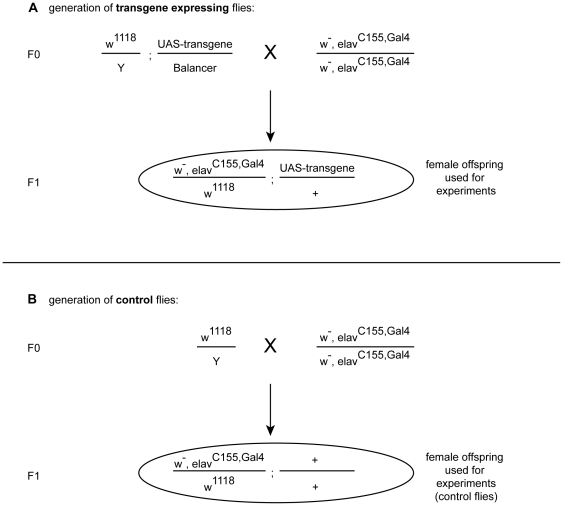
Generation of transgene expressing flies. Males from an UAS-transgene stock were mated with female virgins from the Gal4 line of interest. Female progeny hemizygous for the transgene and Gal4 were selected and used (A). Generation of control flies. Males from w^1118^ were mated with female virgins from the Gal4 line of interest. Female progeny hemizygous for the Gal4 were selected and used. W^1118^ flies are used for control crosses since the P-element insertion of the UAS-transgenes was done in w^1118^ flies (B).

Transgenic flies were generated by crossing male flies from an UAS-stock with female virgin flies from the respective Gal4 driver line. The female offspring used for the study was therefore, hemizygous for the Gal4 driver (Figure 2 A). For generation of control flies, male w^1118^ flies were crossed with female virgin flies of the respective Gal4 driver. W^1118^ flies are used for control crosses since the P-element insertion of the UAS-transgenes was done in w^1118^ flies (Figure 2 B).

### Survival assay

In order to select an optimal expression system we compared the survival of flies expressing hproIAPP in different cell populations. Five commercially available *Gal4* driver lines were used: the pan-neuronal Gal4 driver *P(GawB)elav^C155^* [36], the motor neuron Gal4 driver *P(GawB)D4* [37], the mushroom body Gal4 driver *P(GawB)7B* [38], the glia cell Gal4 driver *P(Gal4)repo* [39], and the photoreceptor Gal4 driver line *P(Gal4-ninaE.GMR)* [40]. Male flies from the *UAS-hproIAPP* transgenic line were crossed with female virgin flies of the respective Gal4 driver and the survival of the female progeny expressing hproIAPP was monitored, and compared with the longevity of the control flies (female progeny derived from crosses of male w^1118^ flies with female virgin flies hemizygous for the respective Gal4 driver (*Gal4*/+)). These survival assays were performed, at 26°C. HproIAPP expression driven by the pan-neuronal Gal 4 driver elav^C155^ lead to a reduced longevity when compared with the appropriate Gal4 control (elav^C155,^ ^Gal4^/+) (n = 100, p<0.001) (Figure 1 C, green and black, respectively). The survival of hemizygous *UAS-hproIAPP* flies (*UAS-hproIAPP* transgenic flies backcrossed with w^1118^ flies, *UAS-hproIAPP/+*) was also monitored. These flies had a significant increased lifespan compared to flies expressing hproIAPP driven by elav^C155^ (n = 100, p<0.001), (Figure 1 C, grey), but they lived also longer than elav^C155,Gal4^/+ control flies.

It is known that the metabolism in *Drosophila* is influenced by temperature. To evaluate how temperature affects the longevity of hproIAPP expressing flies, male *UAS-hproIAPP* flies were mated with female virgin elav^C155,Gal4^ flies at 26°C and the survival of hatched female flies was monitored, at 18°C and 29°C. The significant reduction in lifespan persisted also for flies expressing hproIAPP at 18°C and 29°C compared to *Gal4*/+ control flies (in both cases: n = 100, p<0.001 (Figures S2 B and C).

When expression of hproIAPP was induced by any other of the Gal4 drivers, the flies transgenic for proIAPP had a prolonged survival in comparison to their respective *Gal4*/+ control flies (Figures S3 A–D, right panel). To confirm the expression patterns for the different Gal4 driver lines, flies from each line were mated with flies from a *UAS-nlsGFP* line. Whole-mount brains from progeny flies were stained for GFP and the reaction was visualized by a secondary antibody labelled with Alexa 488. At the same time the brains were counter-stained with an antibody reactive against the neuropil specific protein bruchpilot and visualized by a secondary antibody labelled with Alexa 546. The result thereof verified that all Gal4 lines had the expected expression pattern (Figures S2 A and S3 A–D, left panel).

From the result of the survival assay it can be concluded that the observed decrease in survival of elav^C155,Gal4^>proIAPP flies relates to the expression of hproIAPP and it is not just a consequence due to changes in the genetic background, caused by insertion of the *UAS-hproIAPP* into the fly genome (Figure 1 C, green and grey). The unaltered or increased survival of flies detected after hproIAPP expression driven by the Gal4 driver to motor neurons, mushroom body, glia cells or photoreceptors is not easily explained (Figure S3 E). Surprising was the finding that when hproIAPP expression was driven to the photoreceptors by GMR-Gal4 the median survival was markedly increased compared to the GMR-Gal4 controls. When the amyloid protein transthyretin (TTR) was expressed in the photoreceptors this reduced the survival of the files [30]. In addition, TTR expression lead to a distortion of the eye morphology [30], a finding also reported by Berg et al. [29] and by Crowther et al. after Aβ expression driven to the same cellular location [41]. We have looked for morphological changes of the eye structure after hproIAPP expression, but neither TEM analysis of ommatidia nor SEM analysis of external eye structure showed any morphological deviations. As verified by the GMR driven nlsGFP expression (Figure S3 D, left panel), cells localized to the photoreceptor region synthesize the protein. The variation of median survival days observed in controls (49, 47, 56, 51, and 34, in Figure S3 E) could depend on a variation in genetic background including the degree of Gal4 expression that most likely exists between Gal4 driver lines. Gal4 itself is known to exert neurotoxic effects and it has been reported that expression of high amounts of Gal4 in the eye is associated with apoptotic neuronal loss in Gal4 expressing neurons and accumulation of insoluble Gal4 [42], [43]. The earlier reported reduced longevity observed in response to TTR and Aβ GMR driven expression could depend on a general amyloid mechanism. That is that amyloid fibril formation is a self-driven process, and if initiated the process will continue as long as the precursor is present. This pathway includes also the formation of small cell toxic protein species, known as oligomers. The absence of detectable pathology after hproIAPP expression with 4 out of 5 Gal4 drivers could depend on that aggregation was not initiated. This in turn could depend on low protein expression levels or a rapid clearance of hproIAPP.

To elucidate if the toxic phenotype was restricted to hproIAPP we crossed *UAS-hIAPP* and *UAS-mIAPP* with elav^C155,Gal4^, and survival was monitored at 26°C. It was shown that the survival of flies expressing hIAPP and mIAPP did not differ from that of elav^C155,Gal4^ control flies (p>0.05), and both lived longer than flies expressing hproIAPP p<0.001 (Figures 1 D and E). Median survival days of flies expressing mIAPP (42 days), hIAPP (48 days) or elav^C155,Gal4^/+controls (49 days), did not show any significant difference (for each comparison: n = 100, p>0.05) (Figure 1 F). The absence of reduced survival in flies expressing hIAPP and mIAPP when compared to hproIAPP expressing flies is not related to the level of transgene expression since no significant variation between the four hIAPP lines or between the four mIAPP lines was observed (p>0.05 for hIAPP and p>0.05 for mIAPP).

### Immunological detection of expressed protein

**Figure 3:**
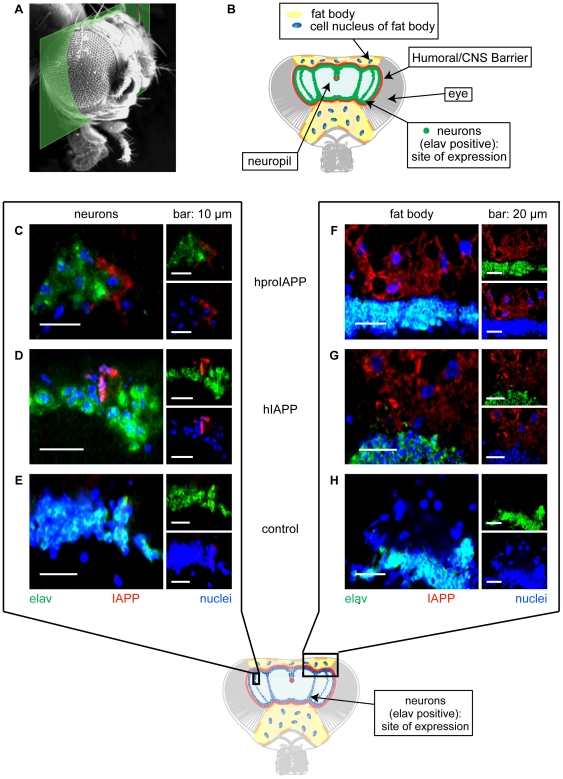
Expressed hproIAPP and hIAPP driven by elav^C155,Gal4^ are secreted from the neurons. (A) The green section highlights the studied area and the carton in (B) is an overview of the most important histological features present in this region. (C, D, E) Inside the humoral-brain-barrier, elav positive neurons are shown (green-light blue) and hIAPP (red) is localized to this area (C, D). (F, G, H) cover an area including both sides of the blood-brain-barrier where the neurons (green-light blue) are present at the bottom part and the fat body in the top part. In (F, G) are the cells in the fat body recognized by IAPP antibodies (red). Immunolabeling of IAPP in the fat body cells demonstrates that hproIAPP (F) and hIAPP (G) are secreted from the neurons, transferred over the humoral-brain-barrier and taken up by cells in the fat body. Cell nuclei are labelled with TOPRO-3 and visualized as blue. Flies were 40 days old.

Based on the results from the lifespan assay we selected the ^elavC155,Gal4^ driver for our model system. The median age for elav^C155,Gal4^>hproIAPP and elav^C155,Gal4^>hIAPP flies were 37 and 48 days respectively (Figure 1 F), and we know from human that the incidence of amyloid increases with age. Therefore, transgenic flies were aged for 40 days, at 26°C. Immunofluorescence analysis was performed on brain sections of elav^C155,Gal4^>hproIAPP and elav^C155,Gal4^>hIAPP flies. The investigated area is indicated in Figure 3 A and areas of interest are highlighted in the cartoon in Figure 3 B. Immunolabeling was performed with a rabbit polyclonal antibody raised against human IAPP, and simultaneous detection of Gal4 positive neurons was performed with an anti-elav specific antibody. IAPP reactivity was detected in some neuronal cells, at site of expected expression as judged by anatomic localization (Figures 3 C and D). In addition to neurons, IAPP reactivity was also detected in the fat body in elav^C155,Gal4^>proIAPP and elav^C155,Gal4^>IAPP flies (Figures 3 F and G). No IAPP labelling occurred at any site in sections from elav^C155,Gal4^/+ control flies (Figure 3 E and H). The head fat body tissue is present outside the humoral-brain-barrier, and no expression of the transgene was expected to take place at this location. This shows that the signal peptide containing transgenic protein was secreted from the neurons and accumulated in the fat body tissue. We would like to point out that the positive staining of the cells in the head fat body in our hproIAPP and hIAPP expressing flies was widespread and not limited to a certain cell population.

**Figure 4:**
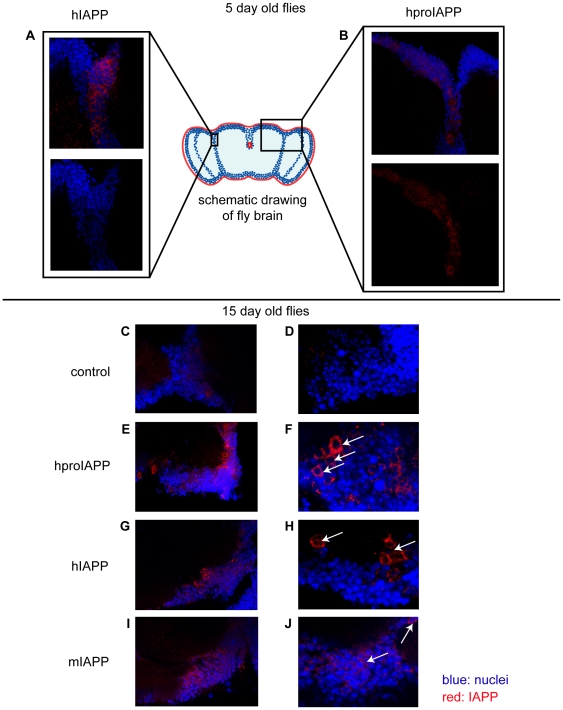
Transgene expression is increased in young flies. The hIAPP, hproIAPP and mIAPP expression driven by the elav^C155, Gal4^ driver was analysed in 5 days (A, B) and 15 days (C–J) old flies. Immunolabeling of whole brain mounts from these younger flies revealed more neuronal immunoreactivity than detected in 30 and 40 days old flies. The depicted immunoreactivity is within the humoral-brain-barrier, the expected site of expression. Immunolabeling was performed with a monoclonal antibody that reacts with human and mouse IAPP. Arrows indicates reactivity.

In contrast to the advanced labelling of the head fat body, only a subset of neurons was found to be immunolabelled for proIAPP and IAPP. It is possible that visible immunoreactivity occurs at sites where proIAPP or IAPP is accumulated while the general absence of reactivity depends on synthesis below the detection level and rapid secretion. The expression of hproIAPP, hIAPP and mIAPP was also studied in younger flies, 5 and 15 days old flies, aged at 26°C. Immunofluorescence analysis was performed on brain sections from elav^C155,Gal4^>hproIAPP, elav^C155,Gal4^>hIAPP and elav^C155,Gal4^>mIAPP flies with a mouse monoclonal antibody reactive against human IAPP. Compared to 40 days old flies, the number of IAPP reactive neurons was higher in 5 days old flies expressing hproIAPP (Figure 4 A), hIAPP (Figure 4 B), and mIAPP and remained increased also in15 days old flies (Figures 4 E–J). No staining could be detected in elav^C155,Gal4^/+control flies (figure 4 C and D).

The result raised the question if levels of Gal4 expression change over time. Males from the, *UAS-nlsGFP* line were mated with female virgins from the elav^C155,Gal4^ line. The expression pattern was studied in dissected whole-mount brains immunolabelled with a primary antibody against GFP and Alexa 488-labelled secondary antibody, at time points 1, 5, 15 and 30 days. The result of this study showed that the elav driven expression of nlsGFP varied over time. Only a limited reactivity was detected in one day old flies. The highest number of reactive cells was present at day 5, and already at day 15 was the number of reactive cells reduced. In brains from 30 days old flies the numbers of reactive cells was almost comparable to that found in 1 day old flies (Figure S4). With this result in mind is the detected low immunolabeling in brains of older flies not surprising.

**Figure 5:**
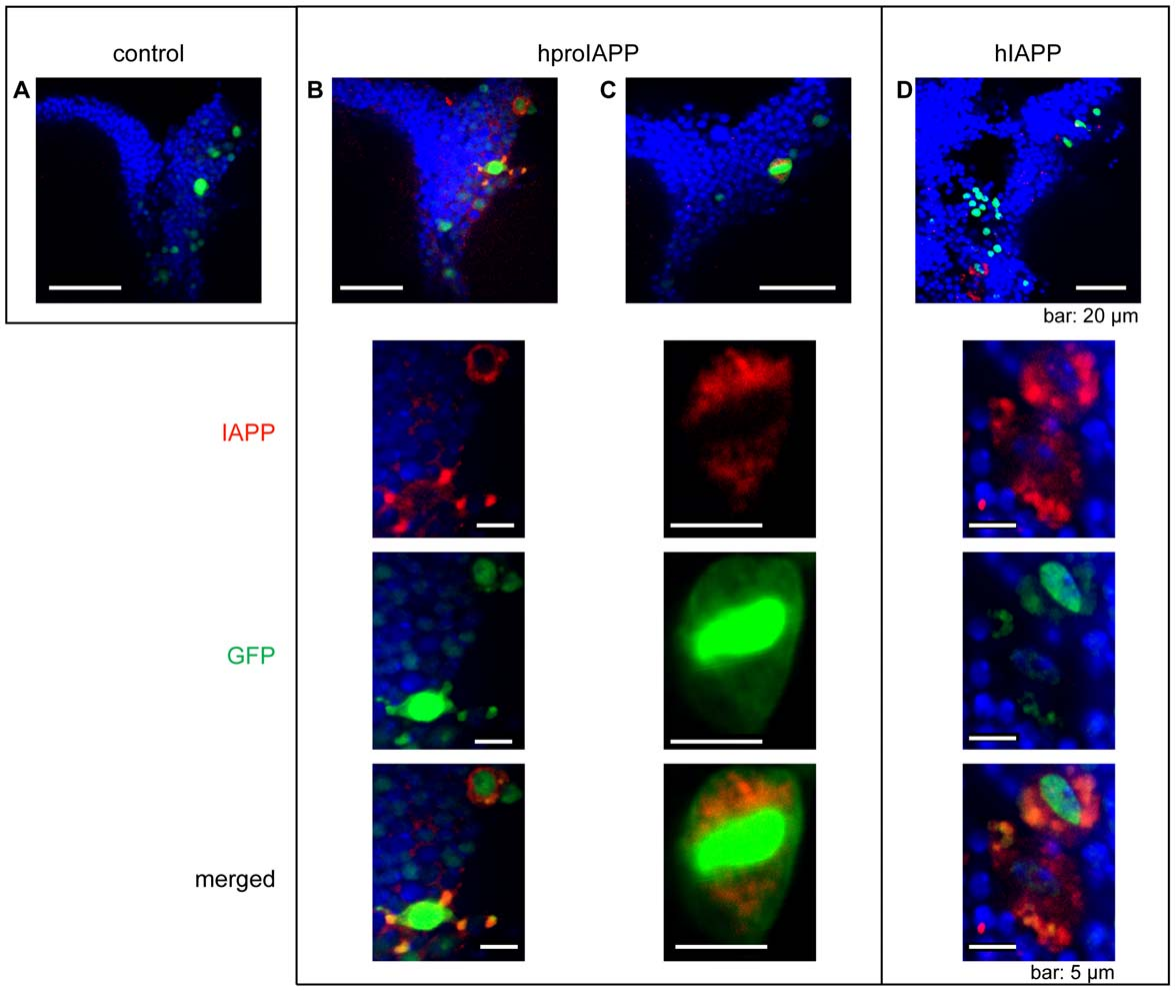
IAPP immunoreactivity is present both intra- and extracellularly in hproIAPP and hIAPP expressing flies. The nls-GFP (green) expression was driven by elav^C155,Gal4^ and included to help to identify the cell nuclei in expressing cells. In control flies (A), IAPP-immunoreactivity is absent as expected. In hproIAPP expressing flies, the IAPP-reactivity (red) is in (B) primarily present extracellularly, separated from the nls-GFP signal, while in (C) the reactivity is intracellular and present adjacent to a nls-GFP labelled nucleus. In hIAPP expressing flies (D), IAPP-reactivity (red) is seen both extra- and intracellularly. The study was performed on dissected whole mount fly brains. To enhance the intensity of the GFP-signal the brains were also labelled with antibodies specific for GFP. The cell nuclei were labelled with TO-PRO-3 and visualized as blue. Flies were 30 days old.

To determine if IAPP reactivity in neurons was present extracellularly and/or intracellularly the cell nuclei of Gal4 containing cells were labelled with GFP. This was obtained by the Gal4 dependent expression of *UAS-nlsGFP* where the nuclear leading sequence (nls) transfers expressed GFP to the cell nucleus (Figure 5 A). In a parallel setup these cells also expressed hproIAPP or hIAPP. After immunolabeling, extracellular IAPP reactivity was detected in both hproIAPP and hIAPP expressing flies (Figures 5 B and D). This reactivity was indicative for aggregates, since non-aggregated peptide would be expected to diffuse. Intracellular IAPP was also present in both hproIAPP and hIAPP flies (Figures 5 C and D). Intracellular IAPP reactive material was sometimes extensive and replaced the main portion of the cytosol, while extracellular aggregates were occational localized to areas free of cell nuclei. This is similar to the pattern seen in hIAPP transgenic mice where intracellular aggregates could replace the cytosol and cause cell death by apoptosis [44].

### Processing of hproIAPP in *Drosophila Melanogaster*

**Figure 6:**
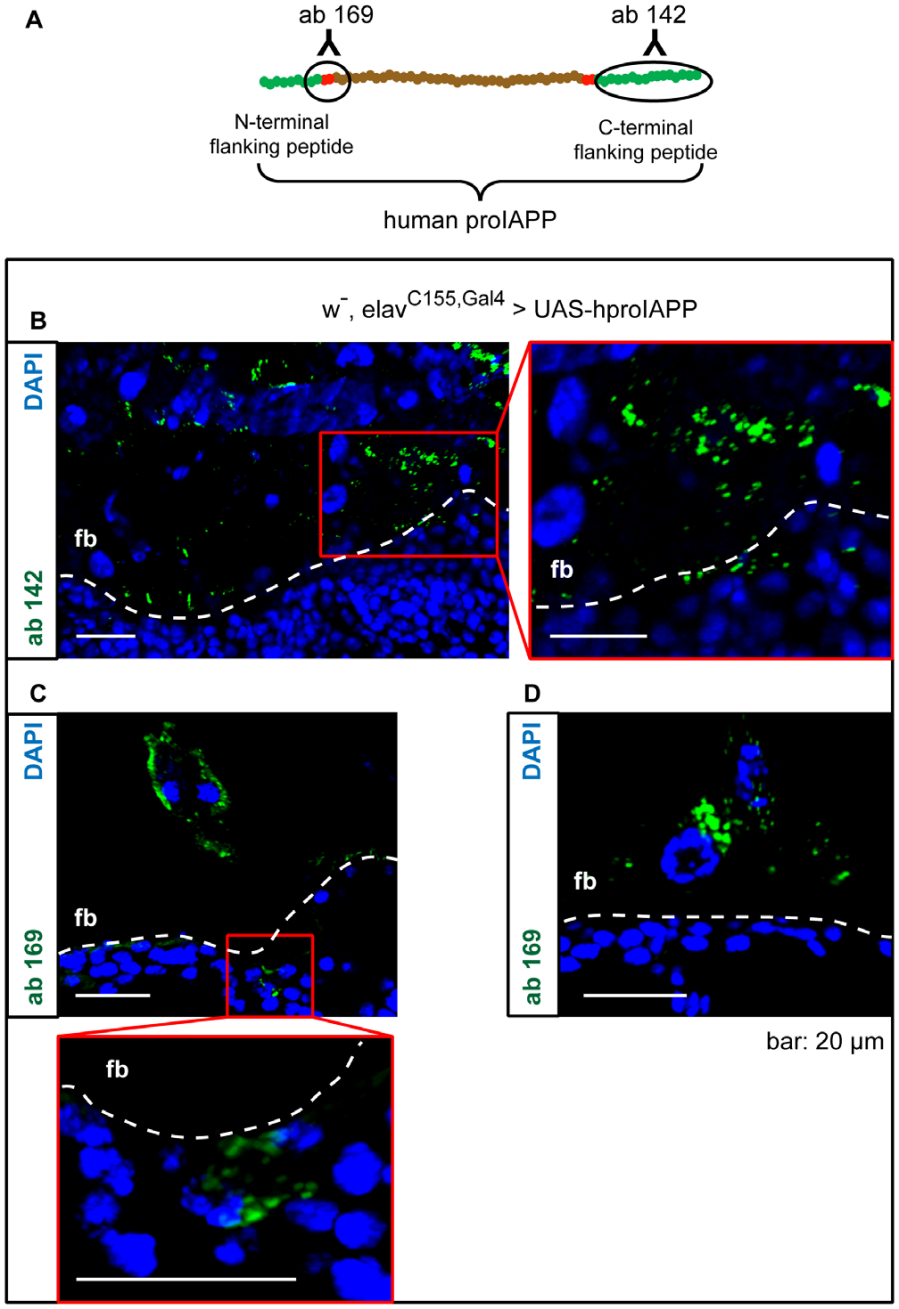
ProIAPP is not processed into IAPP. Sections from hproIAPP flies with the expression driven by elav^C155,Gal4^ were used for immunolabeling with two different antibodies. Antibody A142 is produced against the C-terminal flanking peptide of proIAPP, and antibody A169 is produced against the N-terminal processing site. This antiserum will only bind to proIAPP, unprocessed at this region. The epitopes for A142 and A169 are encircled in (A). Antiserum A142 labels cells inside the humoral-brain-barrier and in the head fat body (B). Antiserum A169 show the same reactivity pattern (C, D). Antiserum-A169 reactivity within the head fat body supports that hproIAPP is not processed by amontillado.

Amontillado is the *Drosophila melanogaster* homologue to the mammalian prohormone convertase 2 (PC2). In mammals, PC2 processing of proIAPP results in the removal of the N-terminal flanking peptide (Figure 1 A). To investigate if hproIAPP was processed in *Drosophila*, we performed immunofluorescence analysis with two different antibodies on head sections from flies with hproIAPP expression driven by the elav^C155,Gal4^ driver. Antibody A-169 is reactive against the N-terminal processing site of proIAPP (Figure 6 A), an epitope only present when the N-terminal flanking peptide is linked to IAPP. This antibody is therefore specific for the N-terminal part of proIAPP [11]. Antibody A-142 reacts with the C-terminal flanking peptide of proIAPP [44] (Figure 6 A), and its reactivity is independent of IAPP. Antibody A-142 and antibody A-169 show reactivity on both sides of the humoral-brain-barrier (Figures 6 B and C). This pattern is comparable to that observed for IAPP (Figures 3 C, D, F and G). The presence of antibody A-169 reactivity in the cytoplasm of head fat body cells proves that proIAPP is not cleaved by Amontillado at the N-terminal processing site (Figures 6 C and D). The reactivity with antibody A-142 in the head fat body region suggests that the C-terminal flanking peptide translocates to the same location as the rest of the molecule and this supports absence of processing.

### Deposited hproIAPP and hIAPP was recognized by Congo red and FTAA

**Figure 7:**
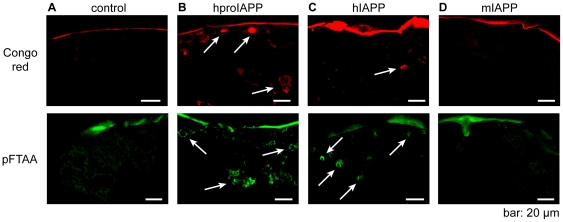
The presence of amyloid deposits is demonstrated after staining with Congo red (upper panel) or with the polyelectrolyte pFTAA (lower panel). Frozen brain sections from control flies and from hproIAPP, hIAPP and mIAPP expressing flies were stained for amyloid. No amyloid could be detected in control flies (A) or in flies expressing the non-amyloid forming mIAPP (D). Amyloid was visualized in hproIAPP (B) and hIAPP (C) transgenic flies in regions corresponding to the expressing neurons, but also to the fat body. Positive staining is indicated by arrows. Congo red staining was viewed at 546 nm with a helium-neon laser and pFTAA staining was viewed at 488 nm with an argon laser. Green and red signal in control and mIAPP expressing flies depends on chitin in the exocytoskeleton. Flies were 30 days old.

To examine if amyloid was formed in *Drosophila* we stained brain sections of aged flies (40 days) with Congo red. As shown in Figures 7 B and C, upper panel, material stained with Congo red was mainly present in the fat body in both hproIAPP and hIAPP expressing flies, with possible minute staining of aggregates in the neuronal area. In addition to Congo red, brain sections were stained for amyloid with pFTAA (lower panel), a recently described amyloid binding luminescent conjugated oligothiophene [45], [46]. Results obtained from pFTAA staining were similar to those obtained with Congo red and with both, aggregates made up by hproIAPP and hIAPP were mainly detected in the fat body region (Figures 7 B and C, lower panel). Though, pFTAA staining was more frequent than Congo red. This may depend on the possible ability of pFTAA to recognize more ¨immaturë aggregates in addition to amyloid [47], [48]. No amyloid like staining could be detected in sections from elav^C155,Gal4^ control flies or mIAPP expressing flies. However, both Congo red and pFTAA show high affinity to the chitin in the exoskeleton, a binding described by Cohen, in 1993 [49].

**Figure 8:**
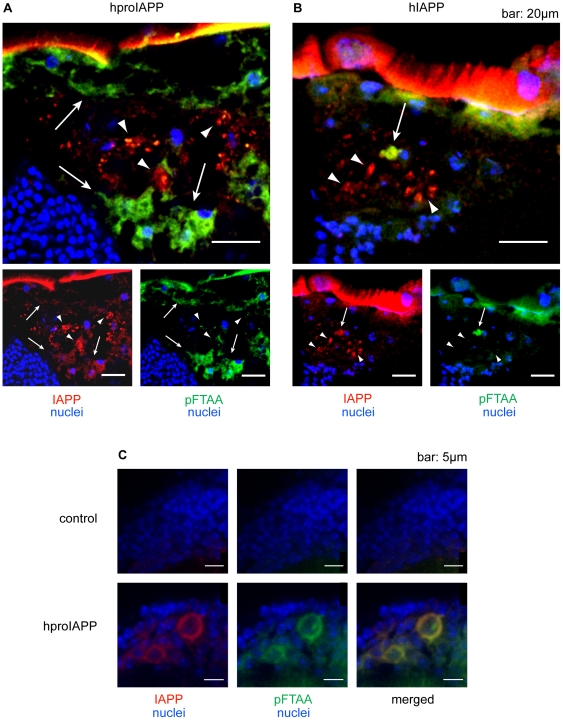
IAPP immunoreactivity co-localizes in part with pFTAA staining. In the fat body region of hproIAPP (A) and hIAPP (B) expressing flies IAPP (red) reactivity is detected in the cytoplasm of the cells in the fat body. The pFTAA (green) staining co-localized with IAPP immunoreactivity is indicated by arrows. IAPP (red) labelled regions not recognized by pFTAA are expected to be in non-fibrilar configuration. These regions are indicated by arrow heads. In the neurons, IAPP (red) and pFTAA (green) co-localize extracellularly (C). This assumption is based on the size of the cell nuclei.

To ensure that pFTAA positive material was made up of IAPP-reactive material, brain sections from flies expressing hproIAPP or hIAPP were stained with pFTAA and immunolabelled with a polyclonal antibody against IAPP. In hproIAPP and hIAPP flies, co-localization of IAPP and pFTAA staining was frequently seen in the fat body (Figures 8 A and B). Areas only labelled with anti IAPP-antibodies represent the peptide in a non-amyloid structure. No area showed pFTAA labelling only (except for the exoskeleton). Co-localization of IAPP and pFTAA staining was also seen inside the humoral-brain-barrier and the reactivity was present both intracellularly and extracellularly (Figure 8 C).

### Ultrastructural analysis of the fat body

The detection of IAPP-immunoreactivity in the fat body of hIAPP and hproIAPP expressing flies in addition to demonstration of structures stained with Congo red, and pFTAA prompted us to have a closer look at the ultrastructure of this tissue. We selected 16 days old transgenic flies since this is before the drop in survival of proIAPP expressing flies (Figure 1 C). Control flies on the other hand had yet another 10 days before their survival started to decline. If alterations occur we should be able to detect them at this stage, and avoid structural changes that might be caused merely by ageing. Frozen sections were treated with ethanol prior to IAPP and pFTAA staining since this treatment solubilizes lipids. In these sections pFTAA (Figure 8 A) and IAPP (Figure 8 B) staining was mainly surrounding the cell nucleus and with thin rims of labelling present between areas earlier occupied by lipid drops.

**Figure 9:**
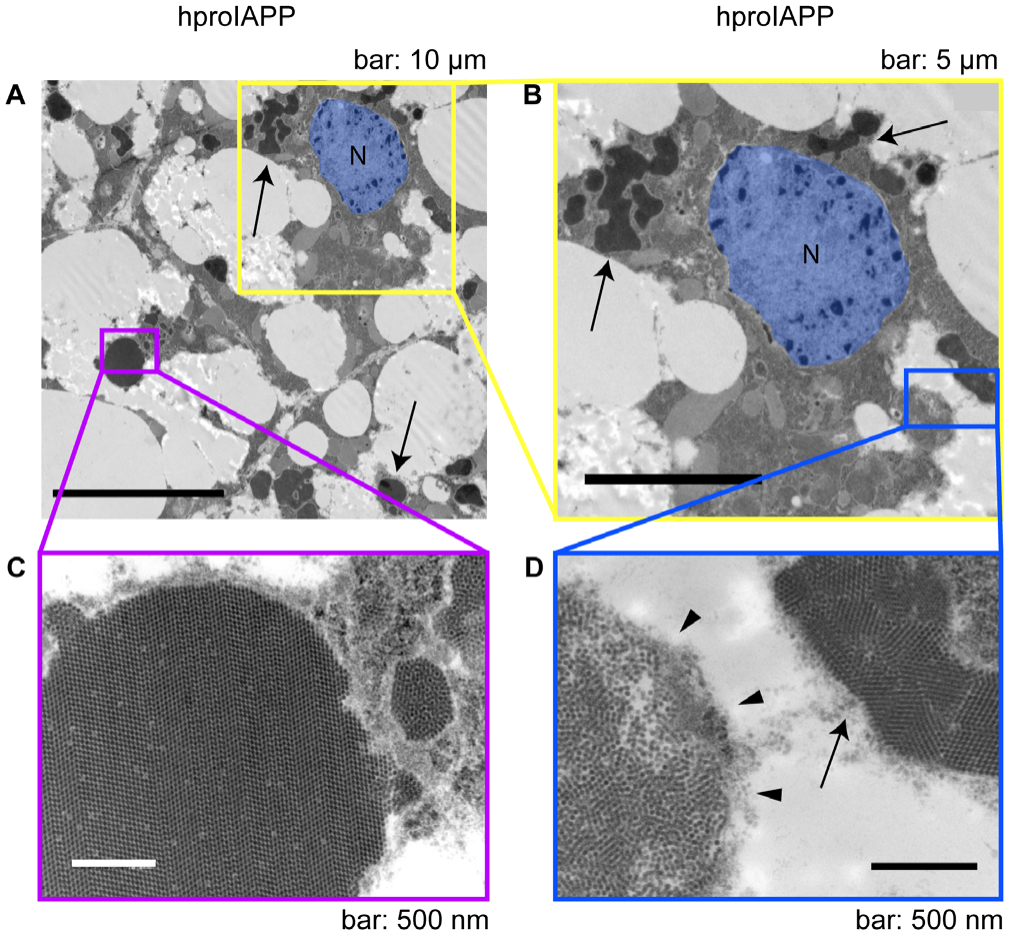
Protein granules accumulate in 30 days old flies with hproIAPP expression driven by elav^C155,Gal4^. In (A, B) an accumulation of electron dense aggregates can be seen. At higher resolutions (C, D) it is shown that these darker aggregated contain a defined pattern. The cell nucleus is pseudo-stained blue (A, B).

In semi thin sections (1 µm), post-fixed with OsO4 to preserve lipids, we observed a difference in distribution of lipid-drops between the different groups of flies. Control and mIAPP expressing flies had several distinct droplets in each fat body cell (Figure S5), while in hproIAPP or hIAPP expressing flies these cells contained large clusters of lipid drops (Figures 9 A and S5). In sections from the latter flies the thickness of the fat body region was slightly increased and could contain two layers of cells and the integrity of the cell borders were lost. In ultra-thin sections from proIAPP and hIAPP transgenic flies we detected irregularly shaped aggregates surrounding the nucleus together with ER and organelles and also between lipid aggregates throughout the cell. Two types of aggregates could be distinguished; a commonly present electron dense (Figures 9 A–D) and a less frequent lighter aggregate (Figures 9 B and D). The dense granules had a size ranging from a few hundred nm to many um in width. Not all aggregates appeared to be membrane encircled and the large difference in size could depend on granule fusion. When viewed longitudinally the aggregates consisted of non-branching straight rope-like structures with a thickness of 15.8 nm. These rods were aligned in parallel with a distance of 5 nm (Figure S6 A). The rod length differed between granules, but they did not exceed 1 µm. In cross-section, each rod had a pentagonal shape with a diameter of 15.8 nm distanced by 5.2 nm (Figure S6 A and B). Each protein granule consisted of groups of rods with different orientations aligned perpendicular to each other (Figures S6 A and C). We performed immunolabeling with monoclonal anti-IAPP antibodies and polyclonal anti-IAPP antiserum, but both failed to react with the aggregates. Instead, only occasional reactivity was detected at the border of the aggregates (Figure S6 C). Since fixation in 2% paraformaldehyde with 0.25% glutaraldehyde and embedding in epon or in hydrophilic resin could block epitopes recognized by the antibodies we performed antigenic retrieval with sodium metaperiodate or H_2_O_2_. This pre-treatment was without success, but it is still most likely that these aggregates arise from proIAPP or IAPP expression, this because immunolabeling with the monoclonal anti IAPP antibody on frozen sections and pFTAA labels the area surrounding the cell nucleus (Figures 8 A and B), and in TEM the highly ordered aggregates were the main structures present at this location. Therefore, we believe that the compact packaging of the molecules hinder recognition by antibodies. It must be stressed that these aggregates were not present in transgenic flies expressing the non–amyloid forming mIAPP or in elav^C155,Gal4^/+ (control) flies. The detected structure is different from what has been described earlier to be found in the fat body cells e.g by Locke et al., [50] in the larvae of the butterfly *Calpodes ethlius* or by Tojo et al., [51] in the pupae of the silkmoth, *Hyalophora cecropia*, and they are also different from the aggregates detected in fat body cells in *Drosophila melanogaster* after expression of double mutant TTR (TTRV14N/V16E) by the GMR driver [52]. In these flies, expression of mutant TTR was driven to the photoreceptors and aggregates were detected in fat body cells of the brain and thorax. These TTR-derived aggregates were determined to have a spherule shape with a 20 nm diameter. The spherules were arranged in a hexagonal pattern and only occasionally short non-branched filaments were detected among the spherules [52]. No similar analysis seems to have been performed on the fat body of Aβ expressing flies. It is possible that proteins are assembled differently in *Drosophila* and mammals, since no amyloid like fibrils could be detected in electron microscopical analysis of sections from 16, 20 or 30 days old flies. Instead, we proposed that the IAPP and pFTAA staining seen in Figures 8 A and B are showing the dense protein aggregate structures despite the absence of immunolabeling in the EM specimens.

Changes of the nuclei morphological were seen in 40 days old hproIAPP and hIAPP expressing flies (Figure S5 B). Here, in some nuclei the normal pattern with heterochromatin and euchromatin had disappeared and was replaced by an evenly dotted pattern. The size of an individual dot exceeded the size of a single nucleosome, but it points to a complete fragmentation of the DNA and initiation of cell death. All studied nuclei in control (Figure S5 A) and mIAPP (Figure S5 C) transgenic flies exhibited unchanged appearance. We did not detect any brain vacuolization in our transgenic flies as it has been described to occur upon TTR expression [29], [30], [41]. Neither of these changes appeared in our flies where the hproIAPP expression was driven to the photoreceptors by the GMR driver.

#### Conclusion

HproIAPP expression driven by the elav^C155,Gal4^ driver causes a significant reduction of the longevity in Drosophila melanogaster. Expression of the amyloidogenic hIAPP and non-amyloidogenic mIAPP did not shorten the longevity and, instead their lifespan was comparable to control flies expressing Gal4 only. Immunolabeling for IAPP revealed reactivity associated with a low number of neurons, but IAPP reactivity was present both intracellularly and extracellularly in such cells. The occurrence of immunoreactivity outside cells indicates presence of protein aggregates. The expression of Gal4 driven by elavC155 driver varies over time, and only few neurons express Gal4 by day 30. This reduction of expression can be one reason for the low toxicity experienced in flies expressing hIAPP. The strong IAPP labeling of the head fat body in hproIAPP and hIAPP expressing flies is indicative for accumulation of peptide at this site. Reactivity in the head fat body of hproIAPP expressing flies with an antibody specific for the N-terminal processing site of hproIAPP show that hproIAPP is not processed by amontillado, the mammalian PC2 homologue. After staining brain sections for amyloid with Congo red and pFTAA, two amyloid specific dyes, amyloid was found mainly in the fat body region with minor deposits in the neurons. The expressed proteins contain a signal peptide and they were expected to be secreted from the neurons. Their accumulation in the cells of the fat body could be explained by transport with the hemolymph followed by sequestration in the fat body cells. The dense granular aggregates present in fat body cells had a rod-like appearance with a pentagonal shape in cross-section. This structure was different from those earlier described, and we suggest that packing of proteins is different in *Drosophila* and mammals. The absence of amyloid or protein aggregates in mIAPP expressing flies strengthens the link between hproIAPP or hIAPP expression and the pathological findings.

## Materials and Methods

### Transgenic constructs and *Drosophila* strains

cDNA of human preproIAPP with the human IAPP signal peptide was cloned into the pUAST vector at the EcoRI/XhoI multiple cloning site to generate *UAS-proIAPP* transgenes. *UAS-hIAPP* and *UAS-mIAPP* transgenes were generated by cloning the respective cDNA into a modified pUAST vector with the signal peptide of human proinsulin. This signal peptide was codon optimized for *Drosophila*. Germ-line transformants were generated in flies with the w^1118^ background by standard technique [53]. The sequence of the transgene was verified by DNA sequencing using the forward primer 5′- CCAGCAACCA AGTAAATCAA CTGC -3′ and the reverse primer 5′- GGCATTCCAC CACTGCTCCC ATT – 3′. These primers bind to regions upstream respectively downstream of the transgene.

The Gal4 driver lines *w^1118^*; *P(Gal4)repo/TM3,Sb*, *w^1118^ (GawB)elav^C155^*, *w^1118^*; *P(Gal4-ninaE.GMR) 12*, *y^1^w^67c23^*; *P(GawB)7B*, and *w^1118^*; *P(GawB)D42* were obtained from Bloomington *Drosophila* Stock Centre, Indiana University, and the UAS-nlsGFP (nuclear leading sequence) line was kindly provided by S. Thor, Linköping University. Expression of the UAS-constructs was induced by crossing the UAS transgenes with the respective Gal4 driver line [54]. Progeny from crosses of the Gal4 lines with w^1118^ flies were used as controls (*Gal4/+*).

All crosses and experiments were performed on flies kept in an incubator (KBWF 720, Binder) with 70% relative humidity, at a 12-hour light/12-dark cycle, at 26°C unless otherwise stated. The flies were cultured on standard food (yeast, syrup, corn meal, and agar). Transgenic flies were transferred to new vials every 2–3 days.

### Quantitative polymerase chain reaction

RNA was extracted from ten, 5 days old female flies from each transgenic group by homogenization in QIAzol lysis reagent (Qiagen, Hilden, Germany) and purification by RNeasy MinElute cleanup columns (Qiagen) according to protocols supplied by the manufacturer. RNA concentrations were determined and 1 µg was used for first strand cDNA synthesis with oligo-dt primer (Fermentas GmbH, St. Leon-Rot, Germany) and the incubation condition 42°C for 1 hour. The reaction was terminated by 5 minutes incubation at 70°C. Real-time PCR reactions were performed and hproIAPP and hIAPP were amplified with forward primer 5′-GCAGCGCCTGGCAAATT and reverse primer 5′-GGTAGATGAGAGAATGGCACCAA, mIAPP was amplified with forward primer 5′-CGCCGGCAAGTGCAA and reverse primer 5′-GCTGGAACGAACCAAAAAGTTT and RP49 (endogenous control) was amplified with forward primer 5′-TGTCCTTCCAGCTTCAAGATGACCATC and reverse 5′-CTTGGGCTTGCGCCATTTGTG. The QT-PCR reaction contained 20 ng cDNA, 300 µmol primer and 15 µl Cyber green (Roche diagnostics GmbH, Mannheim, Germany) and was performed on a Fast Real-Time PCR system (Applied biosystems, Sweden) and the SDS 2.3 software. Relative expression of mRNAs was calculated with the 2^−ΔΔCT^ method, where ΔCt = Ct_(target)_−Ct_(endogenous control)_.

### Life span analysis

Newly hatched flies were collected and females were separated from male flies after 24 hours and divided in groups of 20 flies per vial. Mated female flies, 100–150 from each genotype were aged on standard food and the numbers of surviving flies were scored daily. The flies were transferred to new vials every other day. Statistical analysis was performed using GraphPad Prism 4 (GraphPad Software Inc., La Jolla, CA). Kaplan-Meier survival curves were compared separately for each experimental pair by log rank tests.

### Immunohistochemistry

Aged transgenic flies were carefully decapitated, embedded in Tissue-Tek® O.C.T.™ Compound (Histolab, Gothenburg, Sweden) and stored at −80°C, until used. The embedded heads were brought to −17°C, cut into 10-µm sections and were placed on SuperFrost®Plus slides (Menzel-Gläser, Braunschweig, Germany). The sections were fixed for 10 min in 4% (w/v) paraformaldehyde (PFA) in 0.1 M phosphate buffer with 0.15 M NaCl, pH 7.4 (PBS), and treated in methanol-H_2_O_2_ (20% w/v) to eliminate autofluorescence, as previously described [30]. Unspecific binding was blocked by incubation in PBS with 0.2% Triton X-100 (PBT) and 10% fetal calf serum (FCS) for 1 h, at room temperature. Primary antibodies, monoclonal mouse anti-IAPP (SM1341, Acris Antibodies, Herford, Germany) diluted 1∶100, rat monoclonal anti-elav (7E8A10, Developmental Studies Hybridoma Bank, University of Iowa) diluted 1∶20, polyclonal rabbit antibody against the N-terminal processing site of proIAPP (A169, [11]) diluted 1∶25, polyclonal rabbit antibody against the C-terminal flanking peptide (A142, [44]) diluted 1∶200, and polyclonal rabbit antibody against human IAPP (A133, [11]) diluted 1∶250 in blocking solution were incubated overnight, at 4°C. The following day, sections were rinsed three times 10 min in PBT and the reactivity was detected by Alexa Fluor® 488 (donkey anti-rat, A21208, 1∶1000, Molecular Probes) and Alexa Fluor® 546 (goat anti-rabbit, A11035, 1∶500, Molecular Probes), respectively. Finally, the sections were rinsed three times 10 min in PBT and mounted with PBS/glycerol (1∶1) containing 1 µM To-Pro-3 iodide (T3605, Molecular Probes).

Whole brains of adult *Drosophila melanogaster* were carefully dissected in PBS and fixed in 4% PFA - PBS for 20 min on ice. After fixation, brains were rinsed 3×5 min in PBT and unspecific binding was blocked by incubation in PBT containing 5% FCS, 1 hour at room temperature. The brains were then incubated with primary antibody diluted in blocking solution for 36 hour, at 4°C. Primary antibodies were polyclonal rabbit anti-GFP (TP401, Torrey Pines Biolab) diluted 1∶2000, monoclonal mouse anti-IAPP (SM1341, Acris) diluted 1∶50, and monoclonal mouse anti-bruchpilot (nc82, DSHB) diluted 1∶20. After incubation with primary antibodies, the brains were rinsed three times 40 minutes. Secondary antibodies (Alexa Fluor® 488 goat anti-rabbit, A11008, 1∶500; Alexa Fluor® 546 goat anti-mouse, A11030, 1∶500; Molecular Probes) were diluted in blocking solution and brains were incubated for 24 hours at 4°C followed by 3×40 minutes rinses in PBT at room temperature. Finally, brains were mounted with PBS/glycerol (1∶1).

### Congo red staining

Cryosections (10 µm) were dried onto plus slides and fixed in 95% ethanol for 18 hours, at −20°C, hydrated in 70% ethanol and PBS, at room temperature. Sections were stained for amyloid by 20 minutes incubation in solution A (NaCl-saturated 80% ethanol with 0.01% NaOH) followed by 20 minutes incubation in solution B (solution A saturated with Congo red) (Sigma, Stockholm, Sweden) [55]. Slides were rinsed in absolute alcohol, xylene and mounted with Mountex (Histolab).

### Pentameric formic thiophene acetic acid staining (pFTAA)

Cryosections (10 µm) were dried onto plus slides and fixed in 95% ethanol for 18 hours, at −20°C, hydrated in 70% ethanol and PBS, at room temperature. Sections were stained for amyloid by incubation with 7.5 µM pFTAA diluted in PBS for 20 minutes [45], [46], [47] followed by a short rinses with PBS and water. Slides were mounted with PBS/glycerol (1∶1).

### Confocal analysis

Sections and whole brain specimens were examined with a Nikon eclipse E600 microscope connected to a Nikon C1 confocal unit with argon 488 nm and HeNe 543 nm, and HeNe 633 nm lasers (Nikon, Kawaski, Japan). Sections stained for amyloid with Congo red were studied with confocal microscopy with a HeNe 543 nm laser and sections stained for amyloid with pFTAA were studied with confocal microscopy using an argon 488 nm laser. Pictures were taken with an EZ-C1 digital camera. Images were processed and analyzed with Volocity 4 imaging software (Improvision Inc., Waltham, USA) and Photoshop Elements 4.0 (Adobe).

### Transmission Electron Microscopy (TEM)

Flies were carefully decapitated and the heads were fixed in 2% PFA and 0.25% glutaraldehyde in PBS for 24 hours followed by post-fixation in 2% OsO_4_ and embedded in EPON (Ladd Research Industries, Burlington, USA). Ultrathin sections were placed on nickel grids and contrasted with 2% uranyl acetate in 50% ethanol and Reynolds lead solution. The material was studied at 100 kV in a Jeol 1230 electron microscope (Jeol, Akishima, Japan). Electron micrographs were taken with a Gatan multiscan camera model 791 using Gatan digital software version 3.6.4 (Gatan, Pleasanton, USA).

## Supporting Information

Figure S1 **The survival of four different hproIAPP expressing lines is compared with the survival of control flies, elav^C155,Gal4^/+.** The single transgenic lines hproIAPP#14.2 (green) and hproIAPP#18.5 (purple) showed significant reduction in survival, p.<0.0001 and 0.0175, respectively, while the survival of flies from the hproIAPP#20.4 (blue) was not significantly reduced (p 0.0577). The double-transgenic (orange) line was established by combining hproIAPP#14.2 with hproIAPP#20.4. Flies from this strain show shorter lifespan than control flies (p<0.0001).
(TIF)

Figure S2 **A brain from** ***Drosophila melanogaster*** **where nlsGFP (green) expression is driven by elav^C155,Gal4^.** This is done to visualize the areas for protein expression driven by this driver. The neuropil is labelled with an antibody reactive against the neuropil specific protein bruchpilot (red). Survival of hproIAPP flies was also studied at 18°C (B) and 29°C (C). The expression of hproIAPP shortened the survival at all temperatures, but an increased temperature resulted in shorter lifespan. This is independent of the transgene and it is in line with the knowledge that flies live shorter at higher temperature. The survival of proIAPP expressing flies is presented in green and control flies are shown in black.
(TIF)

Figure S3 **In an initial study we investigated the effects of hproIAPP expression driven by other drivers.** In this study, motor neurons, mushroom body, glia cells (repo-Gal4), and photoreceptor cells (GMR-Gal4) were included. In the left panel of (A, B, C, D), the respective driver was used for expression of nlsGFP to visualize cell regions involved in expression. In the right panel the survival curves are shown for each respective driver. It can be noted that expression of hproIAPP did not cause any reduction of the survival of the flies. With repo-Gal4 and GMR-Gal4 it enhanced survival. The expression of hproIAPP is shown in green and control flies in black. In (E), the median fly survival of the different strains is shown in comparison to flies expressing hproIAPP with the elav^C155,Gal4^ driver.
(TIF)

Figure S4 **The GFP expression driven to neurons by the elav^C155,Gal4^ driver was analysed in 1, 5 15 and 30 days old flies.** The pattern of nlsGFP expression changes over time and only few cells express GFP at day 1. There is a dramatic increase in GFP expression by day 5 and already at day 15 is the GFP positive area decreased. In brains from 30 day old flies is the GFP expression similar to that detected in 1 day old flies. The nlsGFP expression pattern was studied in dissected whole brains after immunolabeling with a primary antibody against GFP that was visualised by an Alexa-488 labelled secondary antibody.
(TIF)

Figure S5 **Electron micrographs of a 40 day old control fly and transgenic hIAPP and mIAPP flies with expression driven by elav^C155,Gal4^.** Shown areas are from the fat body of the head. In addition to protein accumulation in the cytoplasm (shown in Figure 9) a morphological alteration of some nuclei occurs in flies expressing hIAPP (B). In these nuclei, the euchromatin has lost its homogeneity and has instead adopted a dotted pattern. This morphological change of the cell nucleus is also present in flies expressing hproIAPP (not shown), but it is absent in control flies (A) and in flies expressing the non-amyloidogenic mIAPP (C).
(TIF)

Figure S6 **Ultrathin sections from rod-like aggregates present in fat body cells of hproIAPP expressing flies.** Rods with a thickness of 15.8 nm are aligned in parallel and separated by an empty space of 5.2 nm. The individual aggregates consist of both longitudinal and cross sectioned rods and these are arranged perpendicular to each other (A). Cross-sectioned filaments have a pentagonal shape (B). In (C) is IAPP immunoreactivity indicated by arrow heads. The reactivity appears in close association to ends of the rods.
(TIF)

## References

[1] Sipe JD, Benson MD, Buxbaum JN, Ikeda S, Merlini G (2010). Amyloid fibril protein nomenclature: 2010 recommendations from the nomenclature committee of the International Society of Amyloidosis.. Amyloid.

[2] Westermark P, Wernstedt C, Wilander E, Sletten K (1986). A novel peptide in the calcitonin gene related peptide family as an amyloid fibril protein in the endocrine pancreas.. Biochem Biophys Res Commun.

[3] Westermark P, Wernstedt C, O'Brien TD, Hayden DW, Johnson KH (1987). Islet amyloid in type 2 human diabetes mellitus and adult diabetic cats contains a novel putative polypeptide hormone.. Am J Pathol.

[4] Glenner GG, Wong CW (1984). Alzheimer's disease: initial report of the purification and characterization of a novel cerebrovascular amyloid protein.. Biochem Biophys Res Commun.

[5] Gurlo T, Ryazantsev S, Huang CJ, Yeh MW, Reber HA (2010). Evidence for proteotoxicity in beta cells in type 2 diabetes: toxic islet amyloid polypeptide oligomers form intracellularly in the secretory pathway.. Am J Pathol.

[6] Lukinius A, Wilander E, Westermark GT, Engstrom U, Westermark P (1989). Co-localization of islet amyloid polypeptide and insulin in the B cell secretory granules of the human pancreatic islets.. Diabetologia.

[7] Kanatsuka A, Makino H, Ohsawa H, Tokuyama Y, Yamaguchi T (1989). Secretion of islet amyloid polypeptide in response to glucose.. FEBS Lett.

[8] Zhu T, Wang Y, He B, Zang J, He Q (2011). Islet amyloid polypeptide acts on glucose- stimulated beta cells to reduce voltage-gated calcium channel activation, intracellular Ca(2+) concentration, and insulin secretion.. Diabetes Metab Res Rev.

[9] Wang J, Xu J, Finnerty J, Furuta M, Steiner DF (2001). The prohormone convertase enzyme 2 (PC2) is essential for processing pro-islet amyloid polypeptide at the NH2-terminal cleavage site.. Diabetes.

[10] Westermark GT, Steiner DF, Gebre-Medhin S, Engstrom U, Westermark P (2000). Pro islet amyloid polypeptide (ProIAPP) immunoreactivity in the islets of Langerhans.. Ups J Med Sci.

[11] Paulsson JF, Westermark GT (2005). Aberrant processing of human proislet amyloid polypeptide results in increased amyloid formation.. Diabetes.

[12] Huang CJ, Lin CY, Haataja L, Gurlo T, Butler AE (2007). High expression rates of human islet amyloid polypeptide induce endoplasmic reticulum stress mediated beta-cell apoptosis, a characteristic of humans with type 2 but not type 1 diabetes.. Diabetes.

[13] Andersson A, Bohman S, Borg LA, Paulsson JF, Schultz SW (2008). Amyloid deposition in transplanted human pancreatic islets: a conceivable cause of their long-term failure.. Exp Diabetes Res.

[14] Krampert M, Bernhagen J, Schmucker J, Horn A, Schmauder A (2000). Amyloidogenicity of recombinant human pro-islet amyloid polypeptide (ProIAPP).. Chem Biol.

[15] Selkoe DJ (2003). Folding proteins in fatal ways.. Nature.

[16] Westermark P (1972). Quantitative studies on amyloid in the islets of Langerhans.. Ups J Med Sci.

[17] Clark A, Charge SB, Badman MK, de Koning EJ (1996). Islet amyloid in type 2 (non-insulin-dependent) diabetes.. APMIS.

[18] Maloy AL, Longnecker DS, Greenberg ER (1981). The relation of islet amyloid to the clinical type of diabetes.. Hum Pathol.

[19] Sakagashira S, Sanke T, Hanabusa T, Shimomura H, Ohagi S (1996). Missense mutation of amylin gene (S20G) in Japanese NIDDM patients.. Diabetes.

[20] Lee SC, Hashim Y, Li JK, Ko GT, Critchley JA (2001). The islet amyloid polypeptide (amylin) gene S20G mutation in Chinese subjects: evidence for associations with type 2 diabetes and cholesterol levels.. Clin Endocrinol (Oxf).

[21] Yano BL, Johnson KH, Hayden DW (1981). Feline insular amyloid: histochemical distinction from secondary systemic amyloid.. Vet Pathol.

[22] Howard CF (1978). Insular amyloidosis and diabetes mellitus in Macaca nigra.. Diabetes.

[23] Hubbard GB, Steele KE, Davis KJ, Leland MM (2002). Spontaneous pancreatic islet amyloidosis in 40 baboons.. J Med Primatol.

[24] Guardado-Mendoza R, Davalli AM, Chavez AO, Hubbard GB, Dick EJ (2009). Pancreatic islet amyloidosis, beta-cell apoptosis, and alpha-cell proliferation are determinants of islet remodeling in type-2 diabetic baboons.. Proc Natl Acad Sci U S A.

[25] Westermark P, Engstrom U, Johnson KH, Westermark GT, Betsholtz C (1990). Islet amyloid polypeptide: pinpointing amino acid residues linked to amyloid fibril formation.. Proc Natl Acad Sci U S A.

[26] Janson J, Soeller WC, Roche PC, Nelson RT, Torchia AJ (1996). Spontaneous diabetes mellitus in transgenic mice expressing human islet amyloid polypeptide.. Proc Natl Acad Sci U S A.

[27] Verchere CB, D'Alessio DA, Palmiter RD, Weir GC, Bonner-Weir S (1996). Islet amyloid formation associated with hyperglycemia in transgenic mice with pancreatic beta cell expression of human islet amyloid polypeptide.. Proc Natl Acad Sci U S A.

[28] Crowther DC, Page R, Chandraratna D, Lomas DA (2006). A Drosophila model of Alzheimer's disease.. Methods Enzymol.

[29] Berg I, Thor S, Hammarstrom P (2009). Modeling familial amyloidotic polyneuropathy (Transthyretin V30M) in Drosophila melanogaster.. Neurodegener Dis.

[30] Pokrzywa M, Dacklin I, Hultmark D, Lundgren E (2007). Misfolded transthyretin causes behavioral changes in a Drosophila model for transthyretin-associated amyloidosis.. Eur J Neurosci.

[31] Gavin BA, Dolph MJ, Deleault NR, Geoghegan JC, Khurana V (2006). Accelerated accumulation of misfolded prion protein and spongiform degeneration in a Drosophila model of Gerstmann-Straussler-Scheinker syndrome.. J Neurosci.

[32] Byfield P, Turner K, Galante L, Gudmundsson T, MacIntyre I (1969). The isolation of human calcitonin.. Biochem J.

[33] Nelkin B, Rosenfeld K, de Bustros A, Leong S, Roos B (1984). Structure and expression of a gene encoding human calcitonin and calcitonin gene related peptide.. Biochem Biophys Res Commun.

[34] Kitamura K, Sakata J, Kangawa K, Kojima M, Matsuo H (1993). Cloning and characterization of cDNA encoding a precursor for human adrenomedullin... Biochem Biophys Res Commun.

[35] Takei Y, Inoue K, Ogoshi M, Kawahara T, Bannai H (2004). Identification of novel adrenomedullin in mammals: a potent cardiovascular and renal regulator.. FEBS Lett.

[36] Lin DM, Goodman CS (1994). Ectopic and increased expression of Fasciclin II alters motoneuron growth cone guidance.. Neuron.

[37] Yeh E, Gustafson K, Boulianne GL (1995). Green fluorescent protein as a vital marker and reporter of gene expression in Drosophila.. Proc Natl Acad Sci U S A.

[38] Moreau-Fauvarque C, Taillebourg E, Boissoneau E, Mesnard J, Dura JM (1998). The receptor tyrosine kinase gene linotte is required for neuronal pathway selection in the Drosophila mushroom bodies.. Mech Dev.

[39] Sepp KJ, Schulte J, Auld VJ (2001). Peripheral glia direct axon guidance across the CNS/PNS transition zone.. Dev Biol.

[40] Flybase website.. http://flybase.org.

[41] Crowther DC, Kinghorn KJ, Miranda E, Page R, Curry JA (2005). Intraneuronal Abeta, non-amyloid aggregates and neurodegeneration in a Drosophila model of Alzheimer's disease.. Neuroscience.

[42] Kramer JM, Staveley BE (2003). GAL4 causes developmental defects and apoptosis when expressed in the developing eye of Drosophila melanogaster.. Genet Mol Res.

[43] Rezaval C, Werbajh S, Ceriani MF (2007). Neuronal death in Drosophila triggered by GAL4 accumulation.. Eur J Neurosci.

[44] Paulsson JF, Andersson A, Westermark P, Westermark GT (2006). Intracellular amyloid-like deposits contain unprocessed pro-islet amyloid polypeptide (proIAPP) in beta cells of transgenic mice overexpressing the gene for human IAPP and transplanted human islets.. Diabetologia.

[45] Berg I, Nilsson KP, Thor S, Hammarstrom P (2010). Efficient imaging of amyloid deposits in Drosophila models of human amyloidoses.. Nat Protoc.

[46] Aslund A, Sigurdson CJ, Klingstedt T, Grathwohl S, Bolmont T (2009). Novel pentameric thiophene derivatives for in vitro and in vivo optical imaging of a plethora of protein aggregates in cerebral amyloidoses.. ACS Chem Biol.

[47] Nilsson KP, Ikenberg K, Aslund A, Fransson S, Konradsson P (2010). Structural typing of systemic amyloidoses by luminescent-conjugated polymer spectroscopy.. Am J Pathol.

[48] Hammarstrom P, Simon R, Nystrom S, Konradsson P, Aslund A (2010). A fluorescent pentameric thiophene derivative detects in vitro-formed prefibrillar protein aggregates.. Biochemistry.

[49] Cohen E (1993). Chitin synthesis and degradation as targets for pesticide action.. Arch Insect Biochem Physiol.

[50] Locke M, Collins JV (1968). Protein uptake into multivesicular bodies and storage granules in the fat body of an insect.. J Cell Biol.

[51] Tojo S, Betchaku T, Ziccardi VJ, Wyatt GR (1978). Fat body protein granules and storage proteins in the silkmoth, Hyalophora cecropia.. J Cell Biol.

[52] Pokrzywa M, Dacklin I, Vestling M, Hultmark D, Lundgren E (2010). Uptake of Aggregating Transthyretin by Fat Body in a Drosophila Model for TTR-Associated Amyloidosis.. PLoS One.

[53] Spradling AC, Rubin GM (1982). Transposition of cloned P elements into Drosophila germ line chromosomes.. Science.

[54] Brand AH, Perrimon N (1993). Targeted gene expression as a means of altering cell fates and generating dominant phenotypes.. Development.

[55] Puchtler H, Sweat F (1965). Congo red as a stain for fluorescence microscopy of amyloid.. J Histochem Cytochem.

