# Supplementary figures and images for: Drosophila Melanogaster as a Model System for Studies of Islet Amyloid Polypeptide Aggregation
Source: PLoS One. 2011 Jun 14;6(6):e20221. doi: 10.1371/journal.pone.0020221 (PMC3114789; doi:10.1371/journal.pone.0020221)

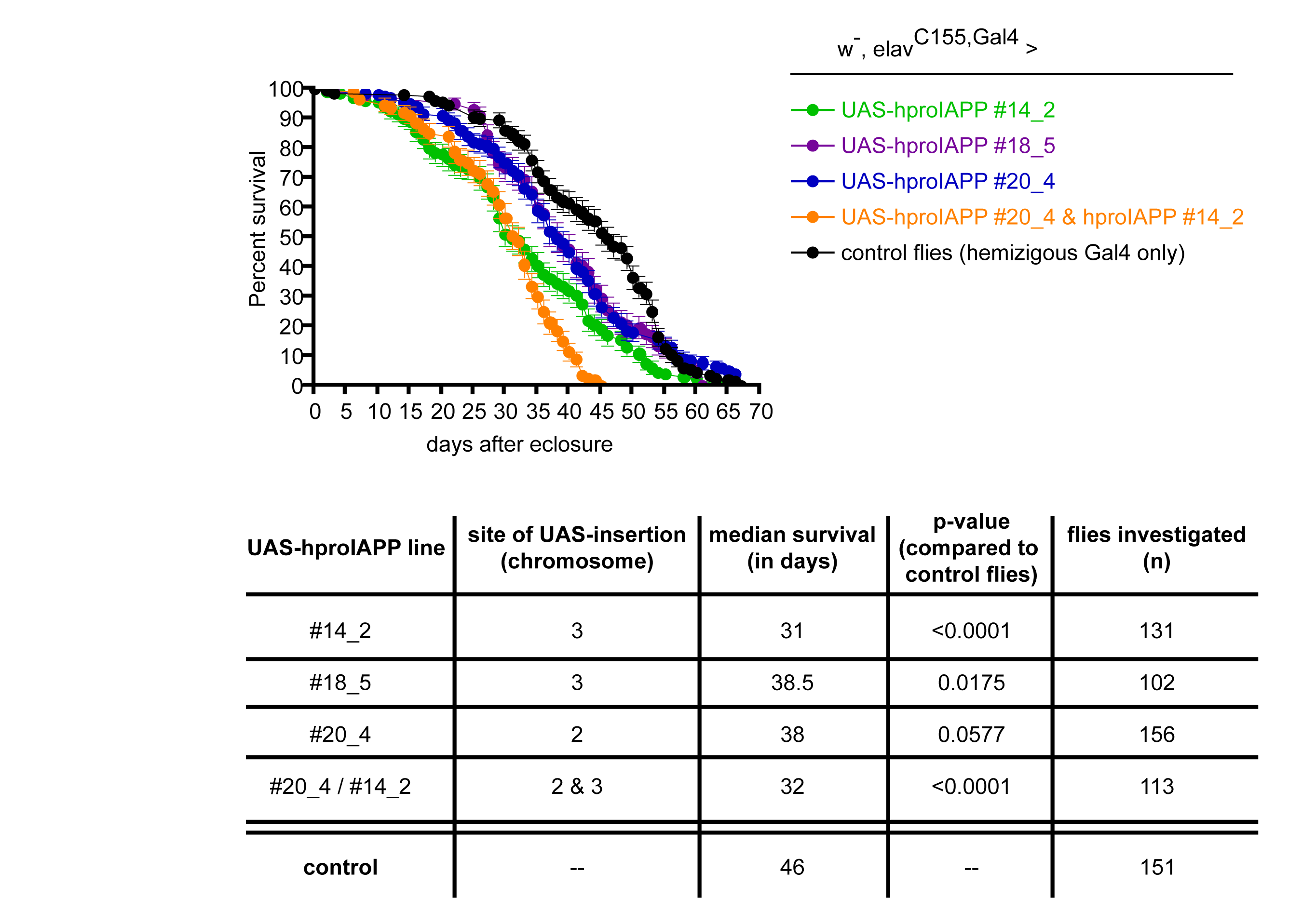

Supplement: Figure S1 — The survival of four different hproIAPP expressing lines is compared with the survival of control flies, elavC155,Gal4/+. The single transgenic lines hproIAPP#14.2 (green) and hproIAPP#18.5 (purple) showed significant reduction in survival, p.<0.0001 and 0.0175, respectively, while the survival of flies from the hproIAPP#20.4 (blue) was not significantly reduced (p 0.0577). The double-transgenic (orange) line was established by combining hproIAPP#14.2 with hproIAPP#20.4. Flies from this strain show shorter lifespan than control flies (p<0.0001). (TIF) [file pone.0020221.s001.tif]

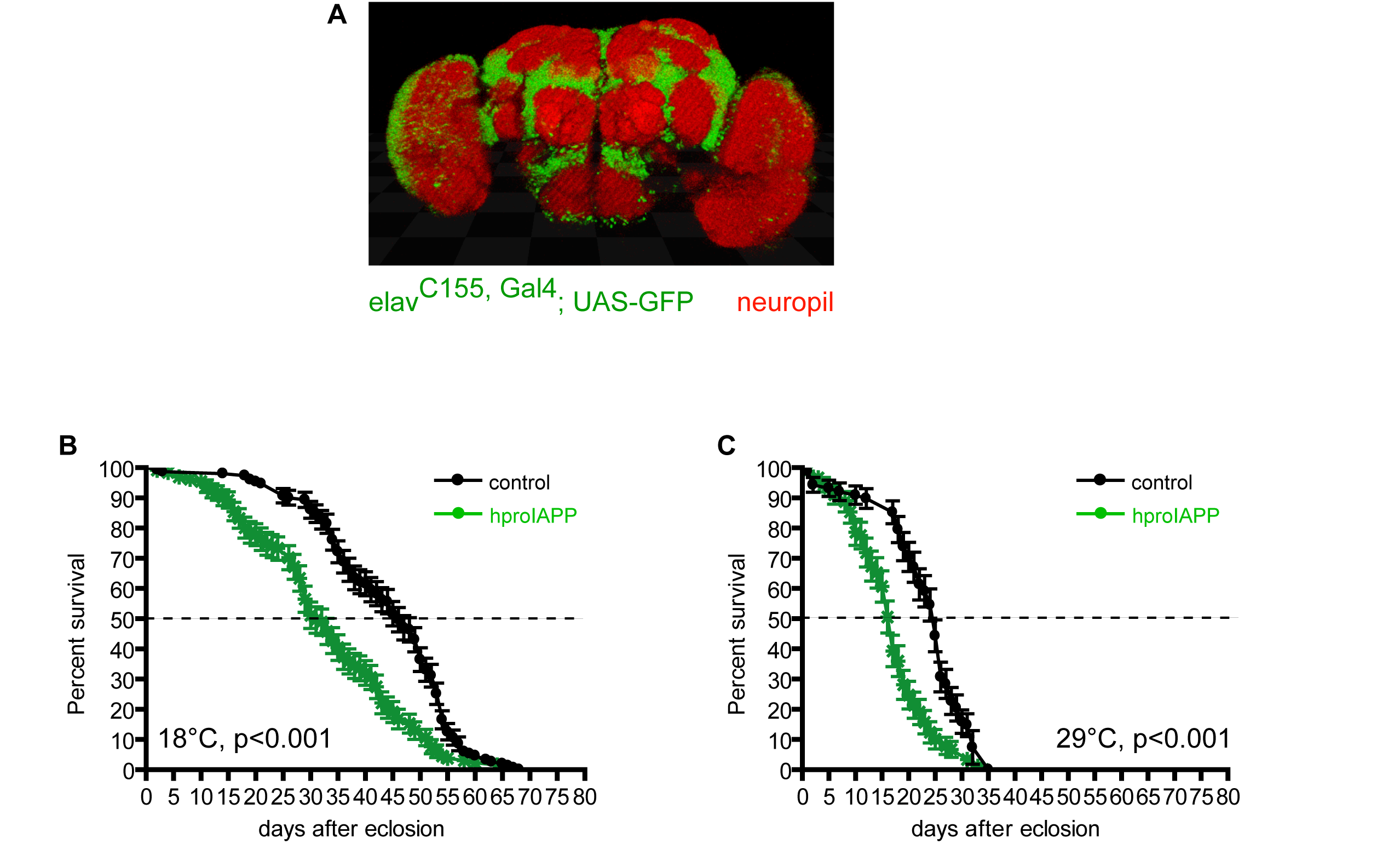

Supplement: Figure S2 — A brain from Drosophila melanogaster where nlsGFP (green) expression is driven by elavC155,Gal4. This is done to visualize the areas for protein expression driven by this driver. The neuropil is labelled with an antibody reactive against the neuropil specific protein bruchpilot (red). Survival of hproIAPP flies was also studied at 18°C (B) and 29°C (C). The expression of hproIAPP shortened the survival at all temperatures, but an increased temperature resulted in shorter lifespan. This is independent of the transgene and it is in line with the knowledge that flies live shorter at higher temperature. The survival of proIAPP expressing flies is presented in green and control flies are shown in black. (TIF) [file pone.0020221.s002.tif]

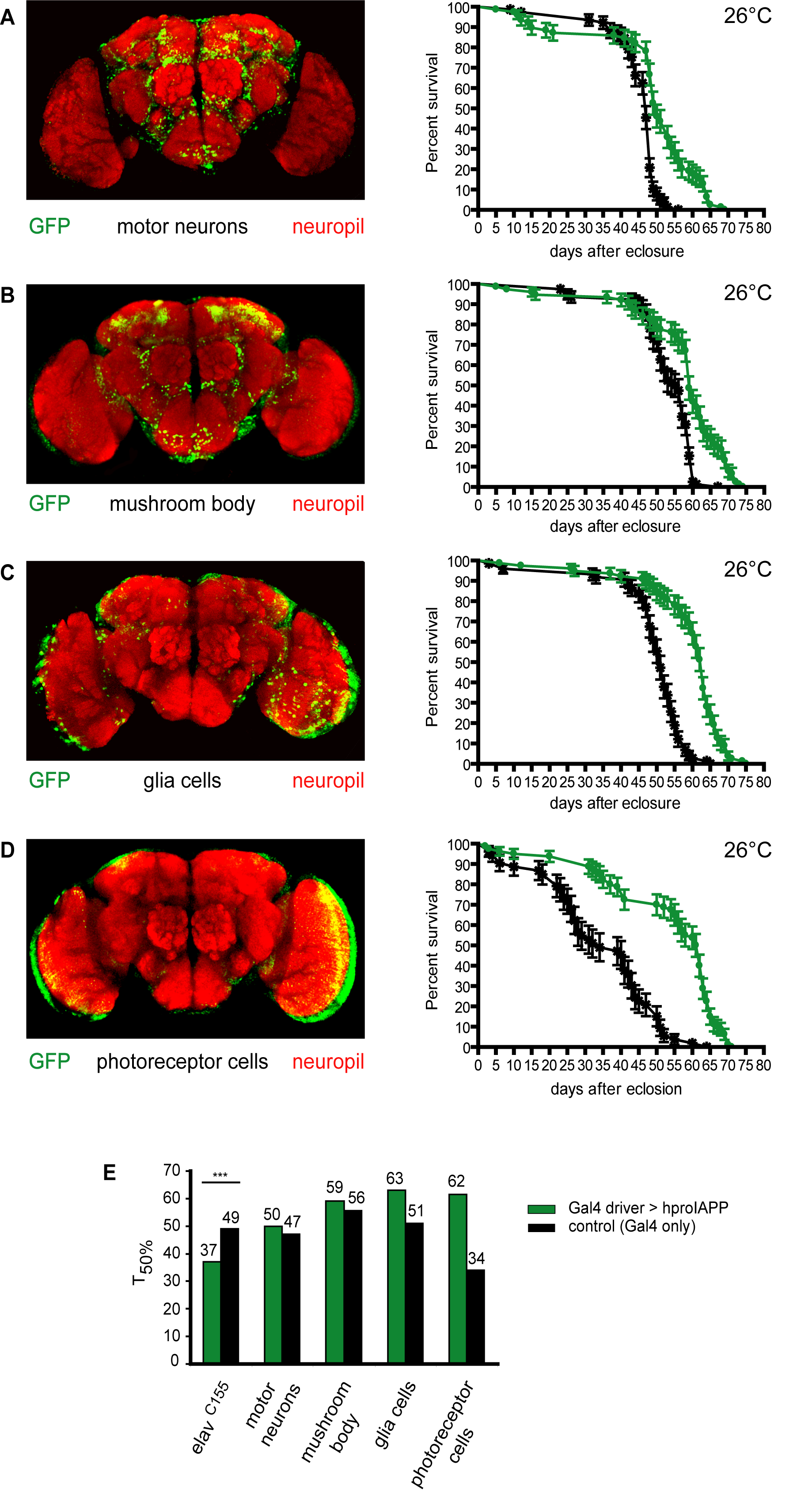

Supplement: Figure S3 — In an initial study we investigated the effects of hproIAPP expression driven by other drivers. In this study, motor neurons, mushroom body, glia cells (repo-Gal4), and photoreceptor cells (GMR-Gal4) were included. In the left panel of (A, B, C, D), the respective driver was used for expression of nlsGFP to visualize cell regions involved in expression. In the right panel the survival curves are shown for each respective driver. It can be noted that expression of hproIAPP did not cause any reduction of the survival of the flies. With repo-Gal4 and GMR-Gal4 it enhanced survival. The expression of hproIAPP is shown in green and control flies in black. In (E), the median fly survival of the different strains is shown in comparison to flies expressing hproIAPP with the elavC155,Gal4 driver. (TIF) [file pone.0020221.s003.tif]

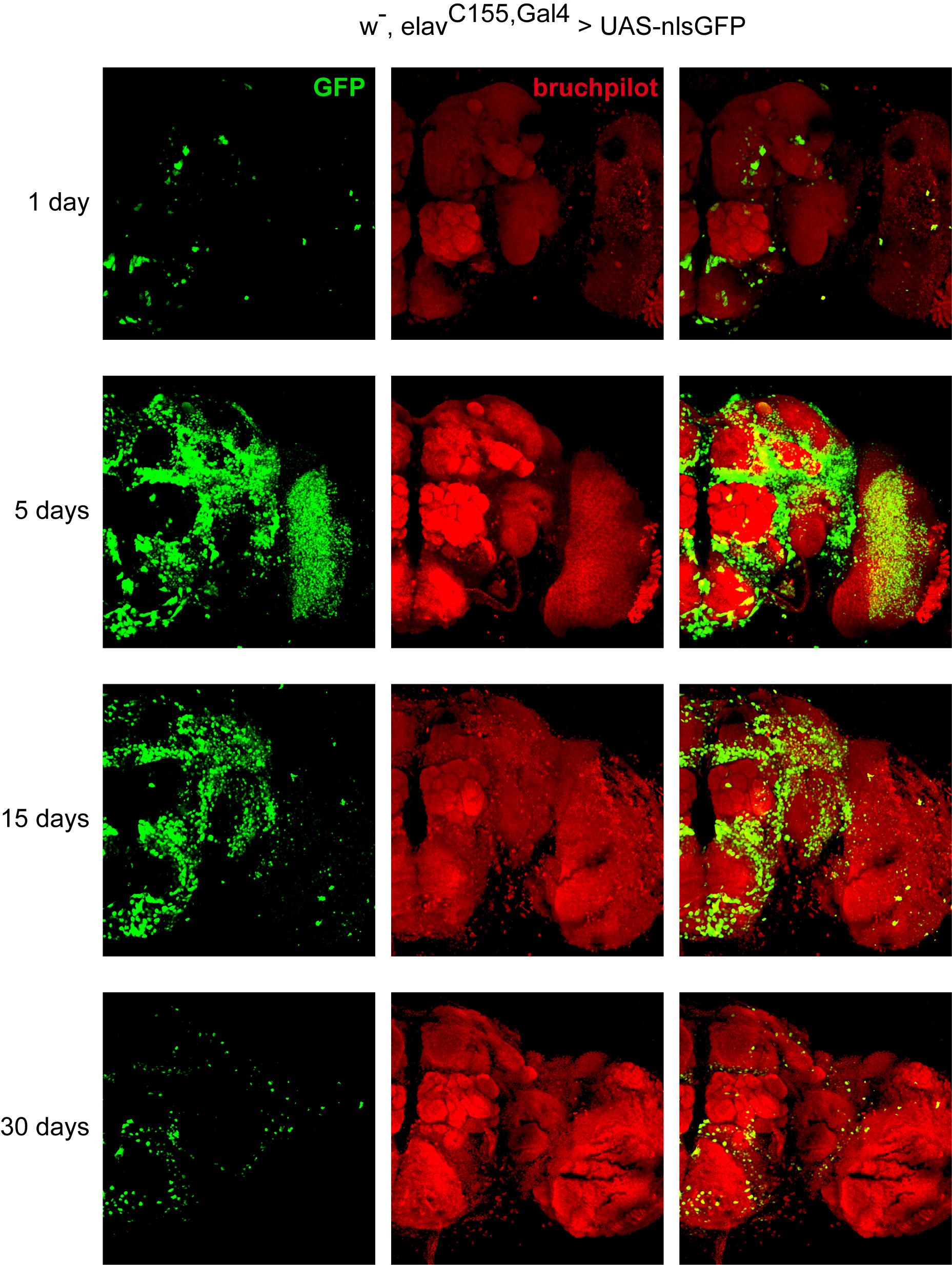

Supplement: Figure S4 — The GFP expression driven to neurons by the elavC155,Gal4 driver was analysed in 1, 5 15 and 30 days old flies. The pattern of nlsGFP expression changes over time and only few cells express GFP at day 1. There is a dramatic increase in GFP expression by day 5 and already at day 15 is the GFP positive area decreased. In brains from 30 day old flies is the GFP expression similar to that detected in 1 day old flies. The nlsGFP expression pattern was studied in dissected whole brains after immunolabeling with a primary antibody against GFP that was visualised by an Alexa-488 labelled secondary antibody. (TIF) [file pone.0020221.s004.tif]

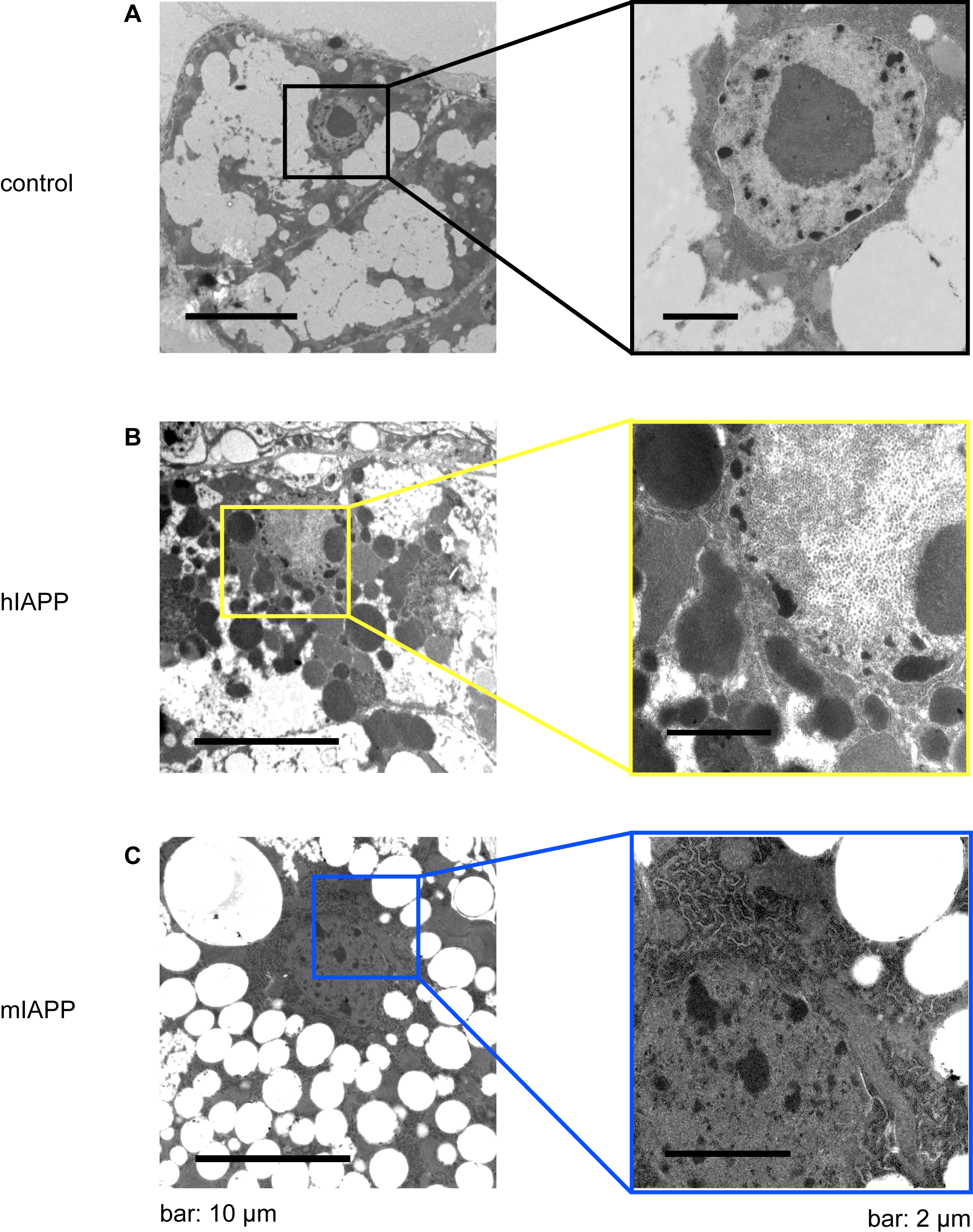

Supplement: Figure S5 — Electron micrographs of a 40 day old control fly and transgenic hIAPP and mIAPP flies with expression driven by elavC155,Gal4. Shown areas are from the fat body of the head. In addition to protein accumulation in the cytoplasm (shown in Figure 9) a morphological alteration of some nuclei occurs in flies expressing hIAPP (B). In these nuclei, the euchromatin has lost its homogeneity and has instead adopted a dotted pattern. This morphological change of the cell nucleus is also present in flies expressing hproIAPP (not shown), but it is absent in control flies (A) and in flies expressing the non-amyloidogenic mIAPP (C). (TIF) [file pone.0020221.s005.tif]

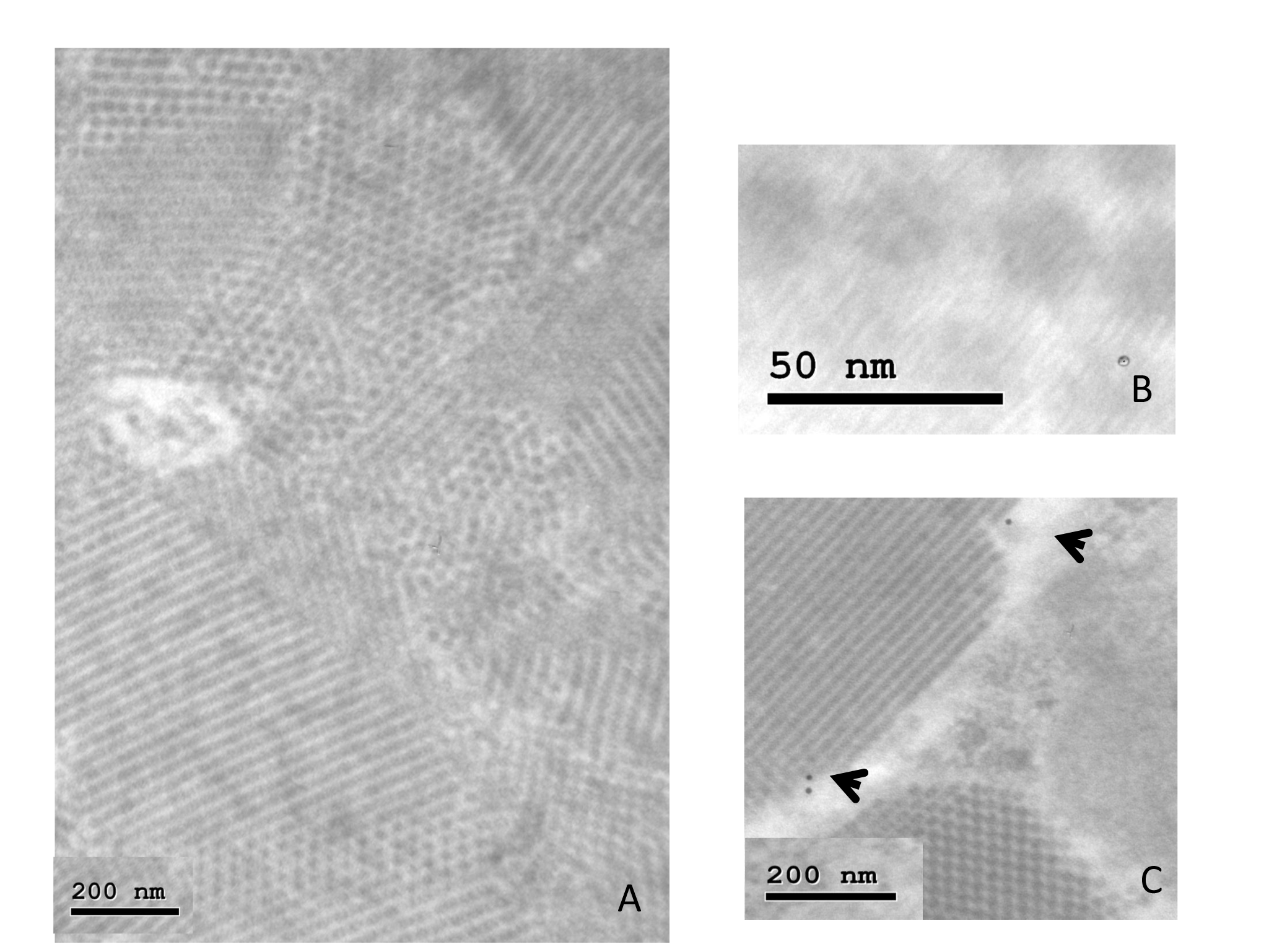

Supplement: Figure S6 — Ultrathin sections from rod-like aggregates present in fat body cells of hproIAPP expressing flies. Rods with a thickness of 15.8 nm are aligned in parallel and separated by an empty space of 5.2 nm. The individual aggregates consist of both longitudinal and cross sectioned rods and these are arranged perpendicular to each other (A). Cross-sectioned filaments have a pentagonal shape (B). In (C) is IAPP immunoreactivity indicated by arrow heads. The reactivity appears in close association to ends of the rods. (TIF) [file pone.0020221.s006.tif]
